# One health disparities and COVID-19

**DOI:** 10.1093/emph/eoab003

**Published:** 2021-02-13

**Authors:** Alma Solis, Charles L Nunn

**Affiliations:** 1 Department of Evolutionary Anthropology, Duke University, Durham, NC 27708, USA; 2 Duke Global Health Institute, Duke University, Durham, NC 27710, USA; 3 Triangle Center for Evolutionary Medicine (TriCEM), Duke University, Durham, NC 27708, USA

**Keywords:** COVID-19, One Health, health disparities, social environment

## Abstract

The global impact of the COVID-19 pandemic has disproportionately affected some communities and populations more than others. We propose that an interdisciplinary framework of ‘One Health Disparities’ advances understanding of the social and systemic issues that drive COVID-19 in vulnerable populations. One Health Disparities integrates the social environment with One Health perspectives on the interconnectedness of human, animal, and environmental health. To apply this framework, we consider One Health Disparities that emerge in three key components of disease transmission: exposure, susceptibility, and disease expression. Exposure disparities arise through variation in contact with COVID-19’s causative agent, SARS-CoV-2. Disparities in susceptibility and disease expression also exist; these are driven by biological and social factors, such as diabetes and obesity, and through variation in access to healthcare. We close by considering how One Health Disparities informs understanding of spillback into new animal reservoirs, and what this might mean for further human health disparities.

**Lay summary:**

One Health focuses on interconnections between human, animal, and environmental health. We propose that social environments are also important to One Health and help illuminate disparities in the coronavirus pandemic, including its origins, transmission and susceptibility among humans, and spillback to other species. We call this framework One Health Disparities.

## INTRODUCTION

The COVID-19 pandemic will be a defining moment in global history due to its impact on all people, regardless of age, gender, or ethnic background. However, the impacts of this crisis will differ greatly among groups of people due to the striking health disparities that have become evident during the pandemic. The National Institute of Minority Health and Health Disparities describes health disparities as preventable diseases that arise due to socially driven underlying systemic issues. Thus, when historians write about the pandemic in the years and decades to come, many will focus on health disparities related to socio-economic status (SES), access to healthcare, education level, environment (rural or urban), politics, and discrimination. 

Here, we integrate perspectives on health disparities with One Health in a novel framework that we call ‘One Health Disparities.’ One Health refers to the interconnections between the health of humans, non-human animals, and the environment [1]. The environment is a key component of One Health; the environment is also central to social determinants of health, where the social environment is key for understanding many health disparities [2]. Thus, we propose that expanding the One Health framework to include aspects of the social environment can help to explain variation in health disparities for zoonotic diseases, such as COVID-19 [2].

**Figure 1.**
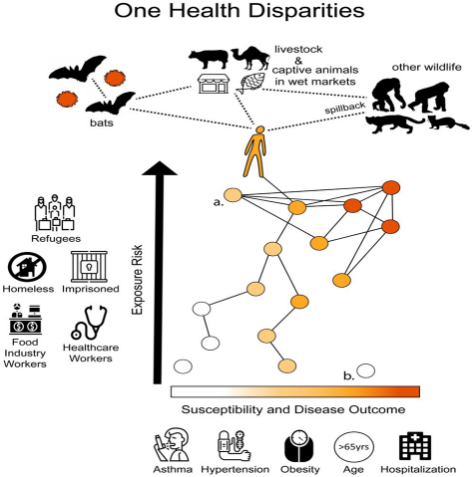
One health disparities. The top of the figure illustrates a standard view of One Health and zoonotic disease in the context of SARS-CoV-2. Bats represent the likely reservoir of SARS-CoV-2, with transmission from bats to humans occurring directly or through bridge hosts (amplification hosts), such as livestock or animals in wet markets. Once in the human population, the virus successfully infected multiple new animal species, including mink, and other species are likely to be susceptible, such as other great apes. The bottom of the figure integrates One Health, social determinants of health, and health disparities within a framework of exposure, susceptibility and disease expression. We represent the social environment and exposure with a social network, revealing a gradient in exposure (top to bottom) that results in exposure disparities. Susceptibility and disease expression are combined here, given that they are difficult to disentangle without experimental studies, and also shown as a gradient (left to right). This results in a gradient of susceptibility and disease expression disparities. The figure shows an expected correlation between exposure and susceptibility-disease expression. However, outliers to this expected correlation will occur. For example, individual (a) is someone who has high exposure but low susceptibility (such as a healthcare worker) and individual (b) is someone who has high susceptibility but low exposure (such as an older person with comorbidities who is able to work from home to reduce exposure).

To explore how One Health Disparities informs understanding of the coronavirus pandemic, we apply it to three critical components of infectious disease transmission and epidemiology: exposure, susceptibility, and disease expression (i.e. morbidity). For transmission of any infectious disease to occur, an individual must be exposed to the infectious agent and susceptible to it; once infected, individuals vary in their expression of disease. One Health Disparities aims to understand how disparities emerge at each of these stages by incorporating perspectives from social determinants of health, One Health, and evolutionary medicine. Figure 1 summarizes the framework that follows, and highlights that many of the social factors show correlated effects on exposure, susceptibility and transmission.

## ONE HEALTH DISPARITIES AND ORIGINS OF SARS-COV-2 AT THE HUMAN-ANIMAL INTERFACE

A major component of One Health research focuses on pathogen transmission at the human-animal interface, including emerging infectious diseases. In the context of One Health Disparities, we can ask: which individuals are most likely to be exposed to a new zoonotic infectious disease, and what are the social drivers of exposure?

A logical starting point to address this question is to consider the source of a new virus—its natural reservoir—and identify which people might be exposed to that reservoir through social factors such as SES, occupation, or ethnic group (Fig. 1). The evolutionary approach of inferring phylogenetic trees is a key approach to investigating animal reservoirs of infectious disease [3]; thus, phylogenies can help identify which groups of people have more contact with those reservoirs. Phylogenetic analyses identified bats as the natural reservoir for SARS-like coronaviruses, with SARS-CoV-1 (the causative agent of SARS) nested within these bat coronaviruses [4]. These findings suggest that individuals who have greater contact with bats will have higher risk of exposure to SARS-CoV-2 (the virus that causes COVID-19 disease).

This appreciation for disparate exposures to the SARS-CoV-1 reservoir informed a study conducted in China. Li et al. [5] collected serological samples from humans in China to identify previous exposure to SARS- related coronaviruses in bats. They also surveyed the same research subjects to determine their exposure to bats, wildlife, and livestock. The serological data confirmed previous infection of bat coronavirus in humans, yet counter to expectations based on the known coronavirus reservoir, the positive subjects reported low contact with bats. Instead, Li et al. [5] identified a high rate of human exposure to other wildlife and livestock as predictors of coronavirus exposure, with the main points of contact involving poultry farming and contact with rodents and shrews living in dwellings. These contacts were also associated with low SES.

The findings from Li et al. [5] may reflect that many coronaviruses utilize bridge hosts (i.e. amplification hosts), which are species that transmit the virus from its natural reservoir to humans [6]. The primary bridge hosts for the SARS- CoV-1 are palm civets (*Paguma larvata*) and for MERS-CoV are dromedary camels (*Camelus dromedarius*; [7]). The bridge host(s) of SARS-CoV-2 is still unknown, but it is hypothesized that SARS-CoV-2 was transmitted to humans through two possible pathways: (1) a bridge-host in animal markets or the wild, and/or (2) through domesticated animals, such as livestock [8]. Understanding disparities in exposure to the bridge host(s) therefore will enable understanding of exposure disparities and inform surveillance and interventions.

Anthropogenic environmental change is also crucial to One Health research, including changes related to increasing reliance on agriculture, increasing limits on land for farming as populations grow, and urbanization and more efficient transportation networks. These transitions are also relevant to understanding SARS-CoV-2 One Health Disparities. In particular, a likely origin point of the virus was in low-income farmers living in rural China, which is associated with land-use change. Yet the farmers’ connections to markets in urban settings, especially those serving as transportation hubs, give emerging diseases opportunities to expand regionally and eventually globally. Thus, a better understanding of how these farmers connect infectious diseases from reservoir or bridge hosts into these transport networks, and the people they interact with when doing so, will likely reveal additional disparities early in the disease outbreak.

## HUMAN-TO-HUMAN TRANSMISSION: DISPARITIES IN SARS-COV-2 EXPOSURE

Once SARS-CoV-2 began spreading in human populations, additional factors led to variation in exposure among humans (Fig. 1). Some environments are riskier for disease transmission than others, whether those are social or physical environments. Importantly, social factors influence the environments in which many people live and work, due to combinations of socioeconomic status, education level, occupations, and social systemic racism.

An important factor in the context of COVID-19 is the ability to avoid high-risk exposure environments, including through social distancing. The inability to socially distance affects some of the world’s most vulnerable populations, including prisoners, the homeless, and refugees. In the workforce, individuals also vary in their ability to follow mitigation strategies, such as working from home, leading to new health disparities in exposure pathways. In 2019, the United States (U.S.) Bureau of Labor Statistics [9] reported that 29% of Americans could work from home and 25% occasionally worked from home. However, only 13% of Hispanic and 18% of Black wage workers were able to work from home. The majority of remote workers had advanced education (a bachelor’s degree or higher) and were high-income salary and wage workers earning above the 75th percentile [9]. In addition to front-line medical workers, the long list of occupations for which working from home is impossible includes cashiers, food preparation and waitresses/waiters, retail salespeople, and janitors.

Variation in wealth and socioeconomic status also influences exposure to risky environments, with a lack of invested capital forcing some people to work, even when that work is not safe in the context of SARS-CoV-2 transmission. Conversely, low SES and unemployment are driving an increase in food insecurity throughout the U.S. and increasing disease exposure risk for people that depend on food banks and pantries for resources [10].

Indoor versus outdoor work represents another important environmental factor that influences variation in exposure to SARS-CoV-2. Occupations that involve outdoor work have reduced risk of exposure, where droplets and aerosols can diffuse more readily by air currents [11]. Thus, a landscaping or farm worker may be at lower risk. However, the increased risk from shared space in vehicles to travel to work sites may offset these benefits. Similarly, policies that require food or services to be provided outdoors may reduce risks for both workers and customers, although this is not possible in all locations or at all times of year.

While much of the world’s focus has been on high-income countries, social distancing in low-income countries also presents challenges due to social and economic factors. Household income is negatively impacted by social distancing , with low-SES workers in low and middle-income countries being less able to sacrifice their livelihoods [12]. In contrast, high-income countries generally have more commonly instituted policies to protect workers from lost jobs or wages.

India provides an example of low-middle income country exposure disparities. The Indian Ministry of Health and Welfare implemented strategies to lower COVID-19 transmission, such as limiting large group gatherings, encouraging people to practice hand and respiratory hygiene, and monitoring common symptoms of COVID-19. These strategies worked best at mitigating transmission in high-income social groups in India, exacerbating exposure-based health disparities [13]. Indeed, Indian states with a high proportion of low-income groups, who have lower SES and lower healthcare nets, have been described as COVID-19 hotspots [13]. In addition, socio-cultural factors also limit mitigation to SARS-CoV-2 in India, such as crowded housing, religious gatherings, and travel using communal services [14].

## DISPARITIES in SARS-COV-2 SUSCEPTIBILITY

Health disparities can also arise following exposure to SARS-CoV-2 through variation in susceptibility, which we define as the probability of becoming infected following exposure to SARS-CoV-2 (Fig. 1). We specifically consider genetic and phenotypic characteristics that may influence susceptibility in humans and our close evolutionary relatives, with the caveat that experimental infection studies have not been conducted for obvious ethical reasons. We also review some social factors and comorbidities that interact with cellular mechanisms associated with susceptibility.

The mutations occurring in SARS-CoV-2 provide some insights to susceptibility because these genetic variants are crucial to the cell receptors needed for entry of the virus into cells. The sites of importance in SARS-CoV-2 are the spike protein (S) and nucleocapsid (N) regions of the genome [15]. Angiotensin-converting enzyme 2 (ACE-2) has a variety of functions, including regulating blood pressure; it is also an important viral receptor that mediates viral binding in SARS-CoV-2 [16]. Among primates, a study revealed gene conservation in ACE-2 in several great apes and suggested that they would be susceptible to SARS-CoV-2 [17]. Another study demonstrated that rhesus macaques can be in infected with SARS-CoV-2 and develop pneumonia [18]. Thus, it seems likely that SARS-CoV-2 can successfully bind to ACE-2 receptors in a wide array of non-human primates and cause illness [17–19].

In humans, variation in expression levels of ACE-2 are well documented, and this is thought to influence susceptibility to SARS-CoV-2 [20, 21]. People with type II diabetes and hypertension exhibit higher expression of ACE-2 [20, 21]. In addition, the elderly and people with chronic lung diseases have higher disease susceptibility to SARS-CoV-2 due to epithelial lung senescent cells, which have high expression levels of ACE-2 [22, 23].

Social environments may also influence susceptibility to SARS-CoV-2. The best evidence for this comes from health conditions that show disparities by SES, race, and ethnicity, such as diabetes, heart disease, and obesity. Given that these health conditions also predict higher risk of SARS-CoV-2, the social factors that drive these conditions should also drive susceptibility to SARS-CoV-2. Similarly, social adversity may influence susceptibility to SARS-CoV-2 infection. For example, lower income and less social integration lead to an increase in chronic diseases, such as liver and kidney disease, diabetes, heart disease, asthma, and stroke [24], which may also increase susceptibility to SARS-CoV-2.

## DISPARITIES in COVID-19 DISEASE EXPRESSION

Exposure and susceptibility are key to explaining the distribution of infections, but it is important to also consider disparities in disease expression (Fig. 1), especially because morbidity and mortality are strongly linked to fitness and the severity of COVID-19 varies markedly among patients. In addition, individuals with greater disease expression (i.e. morbidity) may also have higher viral loads and more effectively transmit SARS-CoV-2 [25], potentially perpetuating the health disparities through contact with friends, co-workers and kin. We acknowledge that some of these disease outcome disparities very likely reflect variation in underlying susceptibility, which was the focus of the previous section; without controlled experiments, it is challenging to disentangle the extent to which observed infections are due to variation in susceptibility or variation in disease severity.

Chronic lung diseases, such as chronic obstructive pulmonary disease (COPD), asthma, pulmonary fibrosis, and lung cancer, appear to increase the severity of COVID-19 [26]. A variety of environmental and social factors can lead to health disparities in these chronic lung diseases. Air pollution impacts 90% of the global population living in urban environments and can lead to an increase in COPD, lung cancer, asthma, and respiratory infections [27]. In these urban settings, however, outdoor air pollution levels and lung health vary considerably based on location; for example, living close to highways increases the likelihood of developing bronchitis and asthma and reduces lung function [28]. Recent evidence suggests that ∼15% of global COVID-19 mortality can be attributed to air pollution [29]. Indoor air pollution also leads to pulmonary disease; for example, lung health is negatively impacted by cooking methods that rely on biomass fuels, such as wood, dried dung, and charcoal [30]. Although the effects of cooking practices on COVID-19 severity are not yet clearly documented, smoking indoors is associated with high-risk of infection and worse health outcomes [31]. In the U.S. pulmonary diseases can also be influenced by targeted retail marketing of menthol cigarettes to Black and Latinx communities [32].

Obesity, diabetes, and vitamin D deficiency also lead to greater COVID-19 severity [33, 34]. These comorbidities also show health disparities that could drive disparities in COVID-19 outcomes. In the U.S. the populations with highest rates of obesity in 2011-2012 were Black and Latinx communities, and American Indian/Alaskan native, Latinx, and Black communities had the highest rates of type II diabetes in the U.S. [35, 36]. Another social determinant of diabetes is education level: people with less than a high school degree had a higher rate of diabetes than those with at least a high school degree [36]. ‘Food deserts’ also contribute to obesity and diabetes, in which access to healthy food is not affordable or readily available in low-income areas. Moreover, Vitamin D deficiency has recently been linked to severe COVID-19 expression and mortality in African Americans [34]. This health disparity is most common in non-Hispanic Black communities, with 84.2% demonstrating insufficient levels of Vitamin D, in comparison to 56.3% of Hispanics and 34.8% of non-Hispanic Whites [37]. Vitamin D deficiency further explains how the environment, social factors, and age contribute to COVID-19 expression, with deficiency more common in fall and winter months, women, obese people, smokers, and older adults (>60 years old) [38].

Political systems that shape the social environment, such as systematic oppression, lead to reduced access to healthcare in historically marginalized communities, though political and healthcare interactions are understudied in evolutionary sciences. This association negatively impacts access to testing or treatment for COVID-19, leading to worse health outcomes. In the United States, this includes immigrant workers. In 1996, the U.S. restricted Medicaid healthcare access to any immigrant for the first five years who came to the country after 1996 [39]. Additionally, non-English speakers report high rates of discrimination in healthcare settings [40]. Overall, 15.2% of Black and 26.7% of Hispanic communities are uninsured, as compared to 9.0% of white Americans [41].

Another gradient in access to healthcare involves proximity to urban centers, which have larger hospitals with more sophisticated equipment, including better-equipped and larger intensive care facilities and access to long-term care. In the U.S., individuals in rural settings have more difficulty accessing healthcare services and may travel two to three times farther to attain preventative and specialized medical care [42]. Similar disparities are seen in large swaths of sub-Saharan Africa, where this disparity in access to care is even more striking, with even fewer medical facilities and fewer physicians and staff to treat severe COVID-19 expression in rural settings [43]. Additional limits to healthcare access in the U.S. include barriers arising from discrimination (race and gender), insurance status, socioeconomic status, and disability status [44].

In some cases, the shutdown associated with social distancing interfered with access to healthcare. A striking example involves the Hualapai tribe, an indigenous group in the southwest of the U.S. The Hualapai tribe attains a majority of their revenue from tourist visits to the Horseshoe Glass Walkway on the south rim of the Grand Canyon. Due to social distancing, the Hualapai Tribe were forced to close this tourist attraction, which left the community without funds to support health clinics. These impacts have been detrimental to indigenous communities. Overall, American Indians and Alaska Natives have 2.8 times more COVID-19 cases and experience a 5-fold higher hospitalization rate, as compared to white counterparts [45].

An enigma has arisen in the case of low-income and low-middle income countries. Although the poorest countries were at first expected to fare worse as SARS-CoV-2 spread globally, the situation has proved more complex. Although reliable data are sometimes hard to come by, these countries appear to have similar levels of infections as seen in higher income countries, but the consequences have been different, with low-income and low-middle income countries requiring fewer hospital beds and less critical care, and experiencing a lower mortality rate [46]. One factor contributing to this pattern may be the younger age structure of low-income and low-middle income countries relative to high-income countries [46]. Given that age is one of the best predictors of disease outcome for COVID-19 [23], this younger age structure of low-income and low-middle income countries may contribute to some reverse disparities in disease outcomes among countries. Although age distribution is a major contributor to having a lower COVID-19 mortality rate in low-income and low-middle income countries, additional factors may also play a role, including lower overall non-communicable disease prevalence compared to high-income countries [43].

Finally, human susceptibility to COVID-19 may also be linked to genetic variants acquired through introgression of Neanderthal DNA to the human lineage, specifically chromosome 3 (SLC6A20), which interacts with ACE-2, and chromosome 9 (9q34), which interacts with the ABO blood group locus [47]. These patterns of ACE-2 expression in some populations with underlying diseases suggest that some genetic factors also explain disease severity, often in combination with environmental and social factors. Given these biological explanations of disease severity, it is important to note that these Neanderthal genetic variants that increase risk are more commonly present in European ancestry, yet mortality is higher in Black, Native American, and Latinx communities. This pattern therefore highlights that social and environmental factors are especially important in driving COVID-19 infections and mortality.

## SPILLBACK: ‘PHYLOGENETIC DISPARITIES’ AND RISKS FOR NEW HUMAN HEALTH DISPARITIES‘’

Returning to One Health’s focus on the human-animal-environment interface, another great concern is that SARS-CoV-2 could enter a new animal species from humans (Fig. 1). This animal could then become a new reservoir of SARS-CoV-2, or its populations could be negatively impacted by COVID-19. While the term ‘spillover’ is commonly used to describe transmission from non-human animals to humans, ‘spillback’ is used to describe transmission from humans to animals, and typically into a new animal host [48].

The discussion from above highlights that many primates are at high-risk for spillback, including other great apes [17, 19]. Thus, spillback has conservation implications, especially for endangered and critically endangered great apes, or could occur in captive populations, including zoos, as seen with tigers at the Bronx Zoo early in the pandemic [49]. Spillback also has economic consequences. For example, mink farms have been negatively impacted by COVID-19. In the Netherlands, mink farmers have culled 17 million minks, and the U.S. Department of Agriculture (USDA) confirmed SARS-CoV-2 infections in mink farms in Utah [50, 51].

In the public health context, it is important to identify which animals can serve as new reservoirs, and in the context of One Health Disparities, which humans will have most contact with those animals, are most likely to become infected, and then develop more severe disease. If SARS-CoV-2 establishes in agricultural animals, this would put farmworkers at greatest risk. It could also create a need for animal vaccines (draining further financial and scientific resources), and could select for new genetic variants that enable the virus to escape vaccination efforts. Although many animals appear to be susceptible to SARS-CoV-2, it is unknown which animals will be competent hosts that are capable of successful disease transmission to humans [52]. For example, ferrets establish upper airway infections, but display inefficient intraspecies transmission, while cats are susceptible to SARS-CoV-2 and display efficient transmission to other cats [53]. A recent study also identified 40+ bat species in North America that may be susceptible to SARS-CoV-2 [54].

The risks considered in this section may vary substantially in low- versus high-income countries. With a larger percentage of the population in a low-income country practicing agriculture, the spread of disease in domesticated animals and people could be particularly challenging, with fewer options for animal vaccination and greater contact between people and domesticated animals.

## CONCLUSIONS AND OUTLOOK

The One Health approach focuses on the connections between human, animal, and environmental health. Here, we argued that social environments are also important to One Health and help to explain health disparities associated with zoonotic disease, ranging from introduction of infectious organisms to human populations, transmission within humans, and possible spillback to other species of wildlife or domesticated animals [55]. Social environments have strong connections to physical environments that also influence health disparities [2, 56]. For example, unhealthy areas in urban ecosystems may have poor sanitation, air pollution, and threatening social environments, while also lacking access to medical care, affordable housing, and healthy food [2]. We applied this framework to the coronavirus pandemic, but it is more broadly applicable to any infectious disease and a wide range of mitigation strategies.

We focused our discussion of One Health Disparities on pathways involving exposure, susceptibility, and disease expression. Social factors affected the original exposure to SARS-CoV-2 due to socioeconomic factors, such as occupation. Social factors also played a role in human-to-human exposure pathways through additional socioeconomic variables that result in limited opportunities to pursue disease mitigation strategies. These exposure disparities are augmented by disparities in the susceptibility to COVID-19 and morbidity in many of these under-privileged communities, including through limited access to medical treatment and increased co-morbidities that are also driven by social factors. Given increased exposure, susceptibility, and expression of COVID-19, this leads to substantial disparities in health outcomes, with a disproportionate number of deaths occurring in marginalized communities.

Reflecting these views, we represented these exposure pathways as a social network in Fig. 1, which also illustrated how social factors influence susceptibility and disease expression. As shown in the figure, common effects of social factors at each stage leads to a correlation in effects in exposure, susceptibility, and disease expression in marginalized communities. Though social networks are an important part of disease transmission [54, 57], the connections between non-human animals, social networks, social environments, and health disparities have yet to be thoroughly modeled using social network analysis and network epidemiology. Identifying the drivers of these disparities in exposure, infection, and disease severity allow public health officers and governments to appropriately allocate resources for more equitable health outcomes. 

## References

[1] Lerner H , Berg C. A Comparison of Three Holistic Approaches to Health: one Health, EcoHealth, and Planetary Health. Front Vet Sci 2017; 4:163. 29085825 10.3389/fvets.2017.00163 PMC5649127

[2] Medicine I. . Rebuilding the Unity of Health and the Environment: A New Vision of Environmental Health for the 21st Century, ed. Hanna K. and Coussens C.. Washington, DC: The National Academies Press, 2001, 96. 22896869

[3] Liu W , Li Y, Learn GH et al Origin of the human malaria parasite Plasmodium falciparum in gorillas. Nature 2010; 467: 420–5. 20864995 10.1038/nature09442 PMC2997044

[4] Yip CW , Hon CC, Shi M et al Phylogenetic perspectives on the epidemiology and origins of SARS and SARS-like coronaviruses. Infect Genet Evol 2009; 9: 1185–96. 19800030 10.1016/j.meegid.2009.09.015 PMC7106296

[5] Li H , Mendelsohn E, Zong C et al Human-animal interactions and bat coronavirus spillover potential among rural residents in Southern China. Biosafety Health 2019; 1: 84–90. 32501444 10.1016/j.bsheal.2019.10.004 PMC7148670

[6] Caron A , Cappelle J, Cumming GS et al Bridge hosts, a missing link for disease ecology in multi-host systems. Veter Res 2015; 46: 83. 10.1186/s13567-015-0217-9 PMC4509689 26198845

[7] Lu G , Wang Q, Gao GF. Bat-to-human: spike features determining ‘host jump’ of coronaviruses SARS-CoV, MERS-CoV, and beyond. Trends Microbiol 2015; 23: 468–78. 26206723 10.1016/j.tim.2015.06.003 PMC7125587

[8] El Zowalaty ME , Järhult JD. From SARS to COVID-19: a previously unknown SARS- related coronavirus (SARS-CoV-2) of pandemic potential infecting humans - Call for a One Health approach. One Health 2020; 9:100124. 32195311 10.1016/j.onehlt.2020.100124 PMC7075990

[9] Labor BoLSUSDo. *JOB FLEXIBILITIES AND WORK SCHEDULES – 2017-2018 DATA FROM THE AMERICAN TIME USE SURVEY.* 2019. p. 4.

[10] Kinsey EW , Kinsey D, Rundle AG. COVID-19 and Food Insecurity: an Uneven Patchwork of Responses. J Urban Health 2020; 97: 332–5. 32504251 10.1007/s11524-020-00455-5 PMC7274516

[11] Prather KA , Wang CC, Schooley RT. Reducing transmission of SARS-CoV-2. Science 2020; 368: 1422–4. 32461212 10.1126/science.abc6197

[12] Thunström L , Newbold SC, Finnoff D et al The Benefits and Costs of Using Social Distancing to Flatten the Curve for COVID-19. J Benefit Cost Anal 2020; 11:179–17.

[13] Nandi A , Balasubramanian R, Laxminarayan R. *Who is at the highest risk from COVID-19 in India? Analysis of health, healthcare access, and socioeconomic indicators at the district level*. medRxiv, 2020: p. 2020.04.25.20079749.

[14] Nandi A , Balasubramanian R, Laxminarayan R. 2020.

[15] Benvenuto D , Giovanetti M, Ciccozzi A et al The 2019-new coronavirus epidemic: evidence for virus evolution. J Med Virol 2020; 92: 455–9. 31994738 10.1002/jmv.25688 PMC7166400

[16] Kuba K , Imai Y, Rao S et al A crucial role of angiotensin converting enzyme 2 (ACE2) in SARS coronavirus-induced lung injury. Nat Med 2005; 11: 875–9. 16007097 10.1038/nm1267 PMC7095783

[17] Melin AD et al Comparative ACE2 Variation and Primate COVID-19 Risk. bioRxiv 2020; 2020.04.09.034967. 10.1038/s42003-020-01370-w PMC7591510 33110195

[18] Shan C , Yao Y-F, Yang X-L et al Infection with novel coronavirus (SARS-CoV-2) causes pneumonia in Rhesus macaques. Cell Res 2020; 30: 670–7. 32636454 10.1038/s41422-020-0364-z PMC7364749

[19] Damas J , Hughes GM, Keough KC et al Broad host range of SARS-CoV-2 predicted by comparative and structural analysis of ACE2 in vertebrates. Proc Natl Acad Sci USA 2020; 117: 22311–22. 32826334 10.1073/pnas.2010146117 PMC7486773

[20] Al-Benna S. Association of high level gene expression of ACE2 in adipose tissue with mortality of COVID-19 infection in obese patients. Obes Med 2020; 19:100283. 32835126 10.1016/j.obmed.2020.100283 PMC7368415

[21] Guo J , Huang Z, Lin L et al Coronavirus Disease 2019 (COVID-19) and Cardiovascular Disease: a Viewpoint on the Potential Influence of Angiotensin-Converting Enzyme Inhibitors/Angiotensin Receptor Blockers on Onset and Severity of Severe Acute Respiratory Syndrome Coronavirus 2 Infection. J Am Heart Assoc 2020; 9: e016219. 32233755 10.1161/JAHA.120.016219 PMC7428639

[22] Leung JM , Yang CX, Tam A et al ACE-2 expression in the small airway epithelia of smokers and COPD patients: implications for COVID-19. Eur Respir J 2020; 55:2000688. 32269089 10.1183/13993003.00688-2020 PMC7144263

[23] Baker SA et al 2020. *Angiotensin-converting enzyme 2 (ACE2) expression increases with age in patients requiring mechanical ventilation*. medRxiv, : p. 2020.07.05.20140467. 10.1371/journal.pone.0247060 PMC7886150 33592054

[24] Snyder-Mackler N , Burger JR, Gaydosh L et al Social determinants of health and survival in humans and other animals. Science 2020; 368:eaax9553. 32439765 10.1126/science.aax9553 PMC7398600

[25] Fajnzylber J , Regan J, Coxen K, The Massachusetts Consortium for Pathogen Readiness et al SARS-CoV-2 viral load is associated with increased disease severity and mortality. Nat Commun 2020; 11:5493. 33127906 10.1038/s41467-020-19057-5 PMC7603483

[26] Association RH. *COVID-19 What You Should Know*. 2020 [cited 2020 11/17/2020]; Available from: https://resphealth.org/covid-19-what-you-should-know/. Accessed 01 November 2020.

[27] Kurt OK , Zhang J, Pinkerton KE. Pulmonary health effects of air pollution. Curr Opin Pulm Med 2016; 22: 138–43. 26761628 10.1097/MCP.0000000000000248 PMC4776742

[28] Wallace J , D’silva L, Brannan J et al Association between proximity to major roads and sputum cell counts. Can Respirat J 2011; 18: 13–18. 21369545 10.1155/2011/920734 PMC3071007

[29] Pozzer A , Dominici F, Haines A et al Regional and global contributions of air pollution to risk of death from COVID-19. Cardiovas Res 2020; 116:2247–2253. 10.1093/cvr/cvaa288 PMC7797754 33236040

[30] Kurmi OP , Lam KB, Ayres JG. Indoor air pollution and the lung in low- and medium-income countries. Eur Respir J 2012; 40: 239–54. 22362845 10.1183/09031936.00190211

[31] Saha J , Chouhan P. Indoor air pollution (IAP) and pre-existing morbidities among under-5 children in India: are risk factors of coronavirus disease (COVID-19)? Environ Pollut 2020; 266: 115250. 32693324 10.1016/j.envpol.2020.115250 PMC7362832

[32] Mills SD , Henriksen L, Golden SD et al Disparities in retail marketing for menthol cigarettes in the United States, 2015. Health Place 2018; 53:62–70. 30055469 10.1016/j.healthplace.2018.06.011 PMC6161357

[33] Popkin BM , Du S, Green WD et al Individuals with obesity and COVID‐19: a global perspective on the epidemiology and biological relationships. Obes Rev10.1111/obr.13128 PMC7461480 32845580

[34] Martín Giménez VM et al Vitamin D deficiency in African Americans is associated with a high-risk of severe disease and mortality by SARS-CoV-2. J Human Hyperten 2020; 10.1038/s41371-020-00398-z PMC7425793 32792611

[35] Spanakis EK , Golden SH. Race/ethnic difference in diabetes and diabetic complications. Curr Diab Rep 2013; 13: 814–23. 24037313 10.1007/s11892-013-0421-9 PMC3830901

[36] Prevention, C.f.D.C.a. 2020*National Diabetes Statistics Report,* 2020.

[37] Taksler GB , Cutler DM, Giovannucci E et al Vitamin D deficiency in minority populations. Public Health Nutr 2015; 18: 379–91. 25112179 10.1017/S1368980014000457 PMC10271268

[38] Orces C , Lorenzo C, Guarneros JE. *The Prevalence and Determinants of Vitamin D Inadequacy among U.S. Older Adults: national Health and Nutrition Examination Survey 2007-*2014. Cureus 2019; 11: e5300–e5300. 31579639 10.7759/cureus.5300 PMC6768617

[39] Ku L , Matani S. Left out: immigrants' access to health care and insurance. Health Aff (Millwood) 2001; 20: 247–56. 11194848 10.1377/hlthaff.20.1.247

[40] Derose KP , Bahney BW, Lurie N et al Review: immigrants and health care access, quality, and cost. Med Care Res Rev 2009; 66: 355–408. 19179539 10.1177/1077558708330425

[41] A. Cohen, A.E.C R , Martinez ME, Terlizzi EP. 2020 Health Insurance Coverage: Early Release of Estimates from the National Health Interview Survey, 2019. National Center for Health Statistics.

[42] Chan L , Hart LG, Goodman DC. Geographic access to health care for rural Medicare beneficiaries. J Rural Health 2006; 22: 140–6. 16606425 10.1111/j.1748-0361.2006.00022.x

[43] Rice BL et al High Variation Expected in the Pace and Burden of SARS-CoV-2 Outbreaks across Sub-Saharan Africa. medRxiv 2020; 2020.07.23.20161208.

[44] Loftus J , Allen EM, Call KT et al Rural-Urban Differences in Access to Preventive Health Care Among Publicly Insured Minnesotans. The Journal of Rural Health: official Journal of the American Rural Health Association and the National Rural Health Care Association 2018; 34:s48–s55. 10.1111/jrh.12235 PMC6069955 28295584

[45] Race/Ethnicity, H.a.D.b 2020. *COVID-19 Hospitalization and Death by Race/Ethnicity Hospitalization and Death by Race/Ethnicity*. [cited 2020 Nov. 17 2020]; Available from: https://www.cdc.gov/coronavirus/2019-ncov/covid-data/investigations-discovery/hospitalization-death-by-race-ethnicity.html. Accessed 25 September 2020.

[46] Walker PGT , Whittaker C, Watson OJ et al The impact of COVID-19 and strategies for mitigation and suppression in low- and middle-income countries. Science 2020; 369: 413–422. 32532802 10.1126/science.abc0035 PMC7292504

[47] Ellinghaus D et al Genomewide Association Study of Severe Covid-19 with Respiratory Failure. N Engl J Med 2020; 383: 1522–1534. 32558485 10.1056/NEJMoa2020283 PMC7315890

[48] Kelly DW , Paterson RA, Townsend CR et al Parasite spillback: a neglected concept in invasion ecology? Ecology 2009; 90: 2047–56. 19739367 10.1890/08-1085.1

[49] McAloose D et al From People to Panthera: Natural SARS-CoV-2 Infection in Tigers and Lions at the Bronx Zoo. mBio 2020; 11:10.1128/mBio.02220-20 PMC7554670 33051368

[50] Molenaar RJ , Vreman S, Hakze-van der Honing RW et al Clinical and Pathological Findings in SARS-CoV-2 Disease Outbreaks in Farmed Mink (Neovison vison). Vet Pathol 2020; 57: 653–657. 32663073 10.1177/0300985820943535

[51] Dyer O. Covid-19: Denmark to kill 17 million minks over mutation that could undermine vaccine effort. BMJ 2020; 371:m4338. 33168526 10.1136/bmj.m4338

[52] Gervasi SS , Civitello DJ, Kilvitis HJ et al The context of host competence: a role for plasticity in host-parasite dynamics. Trends Parasitol 2015; 31: 419–25. 26048486 10.1016/j.pt.2015.05.002 PMC4567474

[53] Shi J , Wen Z, Zhong G et al Susceptibility of ferrets, cats, dogs, and other domesticated animals to SARS-coronavirus 2. Science (New York, N.Y.) 2020; 368: 1016–1020. 10.1126/science.abb7015 PMC7164390 32269068

[54] Olival KJ , Cryan PM, Amman BR et al Possibility for reverse zoonotic transmission of SARS-CoV-2 to free-ranging wildlife: a case study of bats. PLoS Pathogens 2020; 16: e1008758–e1008758. 32881980 10.1371/journal.ppat.1008758 PMC7470399

[55] Santini JM , Edwards SJL. Host range of SARS-CoV-2 and implications for public health. Lancet Microbe 2020; 1: e141–e142. 32835344 10.1016/S2666-5247(20)30069-0 PMC7302768

[56] Schell CJ , Dyson K, Fuentes TL et al The ecological and evolutionary consequences of systemic racism in urban environments. Science 2020; 369:eaay4497. 32792461 10.1126/science.aay4497

[57] Read JM , Eames KTD, Edmunds WJ. Dynamic social networks and the implications for the spread of infectious disease. J Royal Soc Interf 2008; 5: 1001–1007. 10.1098/rsif.2008.0013 PMC2607433 18319209

